# Surveillance of *Culex* spp. vectors and zoonotic arboviruses at a zoo in the United Kingdom

**DOI:** 10.1016/j.heliyon.2024.e26477

**Published:** 2024-02-15

**Authors:** Arturo Hernandez-Colina, Nicola Seechurn, Taiana Costa, Javier Lopez, Matthew Baylis, Jenny C. Hesson

**Affiliations:** a Department of Livestock and One Health, Institute of Infection, Veterinary and Ecological Sciences, University of Liverpool, Leahurst Campus, Chester High Road, Neston, Cheshire, CH64 7TE, UK; b North of England Zoological Society (Chester Zoo), Caughall Road, Chester, CH2 1LH, UK; c Department of Medical Biochemistry and Microbiology/Zoonosis Science Centre, Uppsala University, Box 582, SE-751 23, Uppsala, Sweden; d Department of Veterinary Anatomy, Physiology and Pathology, Institute of Infection, Veterinary and Ecological Sciences, University of Liverpool, Leahurst Campus, Chester High Road, Neston, Cheshire, CH64 7TE, UK; e The Veterinary Pathology Group, Horner Court, 637 Gloucester Road, Horfield, Bristol, BS7 0BJ, UK; f Health Protection Research Unit in Emerging and Zoonotic Infections, University of Liverpool, UK; g Biologisk Myggkontroll, Nedre Dalälvens Utvecklings AB, Gysinge, Sweden

**Keywords:** Alphavirus, Avian disease, Culicidae, Flavivirus, Xenomonitoring, Zoonotic disease, UK

## Abstract

The emergence of several zoonotic mosquito-borne pathogens in Europe, including West Nile virus, Sindbis virus and Usutu virus, has emphasised the importance of consistent surveillance. Considerable fieldwork effort is usually needed to detect low-prevalence pathogens in mosquitoes and screening vertebrate hosts and reservoirs is rarely done simultaneously with mosquito sampling. Zoological gardens offer an opportunity for the surveillance of pathogens, mosquitoes, hosts, and reservoirs concurrently; thus, the aim of this study was undertaking integrated surveillance for mosquito-borne pathogens of wild birds and mosquitoes in Chester Zoo (Cheshire) in the United Kingdom. Mosquitoes were collected in September 2020 and tested for zoonotic bird-hosted arboviruses (i.e., West Nile virus, Usutu virus and Sindbis virus) using RT-qPCRs. Of the 3316 mosquitoes trapped, 98% were identified as *Culex* spp. The average minimum prevalence of the viruses found in the literature was used to calculate the sample size needed for detecting these viruses with 99% confidence. The testing of 2878 *Culex* females found no evidence of presence of the three viruses. Significant differences were found in mosquito abundance per sampling site and collection date; furthermore, important sources of immature and resting mosquitoes were found near aviaries. Eighteen wild birds belonging to 11 species were found dead in the zoo from May to December 2020 and were RT-qPCR tested for West Nile virus and Usutu virus; all samples resulted negative for viral infection. It is unlikely that these viruses were present in the zoo during the sampling period; however, since they circulate in Europe and Usutu virus has been isolated in the United Kingdom and may overwinter here, continued monitoring of mosquitoes and wild birds is recommended as virus introduction and dissemination are possible. This study highlights the importance of regular and integrated arboviral surveillance of zoonotic pathogens in zoos providing baseline information to that end.

## 1 Introduction

Several mosquito-borne infections affecting humans and animals are considered autochthonous or are emerging and disseminating throughout Europe, such as endemic transmission of West Nile virus (WNV) in the Netherlands, outbreak of Sindbis virus (SINV) in Finland, and the isolation of Usutu virus (USUV) in the United Kingdom (UK) [[1], [2], [3], [4]]. As the introduction and dispersal of mosquito-borne pathogens is linked to the presence of competent vectors, vector surveillance guidelines have been developed [5]. The need for sustainable and standardised surveillance in vectors, animals and humans is well recognised [6]; however, resource limitations often prevent extensive monitoring of mosquitoes and wildlife reservoirs. Zoological gardens contain a unique combination of native and exotic species of plants and animals and offer an opportunity for integrated vector and host surveillance to detect transmission of vector-borne pathogens [7].

The multispecies coexistence in zoos could lead to unusual interactions among hosts and pathogens and represents an infection risk to zoo animals, indigenous species, and humans [7]. Relevant zoonotic arboviruses have been detected in zoological gardens; for instance, the first fully sequenced strain of WNV in North America was isolated from a captive flamingo [8], and the first human cases of WNV in the Americas were associated with an outbreak of WNV at the Bronx Zoo/Wildlife Conservation Park [9]. The recent introduction and possible overwintering of USUV in the UK was detected during wild bird surveillance at London Zoo [4,10]. In Germany, the first detection of WNV involved birds from a zoological collection, and mosquito viral screening has demonstrated the presence of SINV among other mosquito-borne pathogens [11,12]. In Austria, captive great grey owls (*Strix nebulosa*) have died from USUV infection [13]. Serological evidence of WNV and USUV infections in zoo birds and mammals has been reported in France [14], including the co-infection of both viruses affecting captive wild birds [15]. Moreover, zoos can also serve as early-detection locations as demonstrated in Slovenia, where the serological testing of animals from the zoological collection revealed the presence of WNV and USUV ahead of the human, domestic animal, and wild animal case reports [16].

Mosquitoes of the genus *Culex* have a central role in the transmission of several arboviruses as well as avian malaria, which is a health and conservation threat for susceptible birds in zoos like penguins [17,18]. The main European vectors for WNV are *Cx. modestus*, *Cx. perexiguus* and *Cx. pipiens* s.l. [19], and *Cx. pipiens* s.l. and *Cx. torrentium* are additionally competent vectors for USUV, SINV and WNV [[20], [21], [22]]. Avian malaria parasites can complete their sporogony in *Cx. pipiens* s.l. in the wild and in lab settings, thus being their most likely vector [23,24]. This makes *Culex* spp. a critical target for mosquito surveillance.

To increase the knowledge of vector-borne pathogens and mosquito ecology at Chester Zoo, monitoring of mosquitoes began in 2017 and has continued since. Here we present the results of the mosquito and avian surveillance effort from 2020, where we investigated if the zoonotic bird-hosted arboviruses circulating in Europe (WNV, USUV and SINV) were present and could affect the captive birds and potentially represent a risk to people.

## 2 Materials and methods

### 2.1 Mosquito collections

**Fig. 1:**
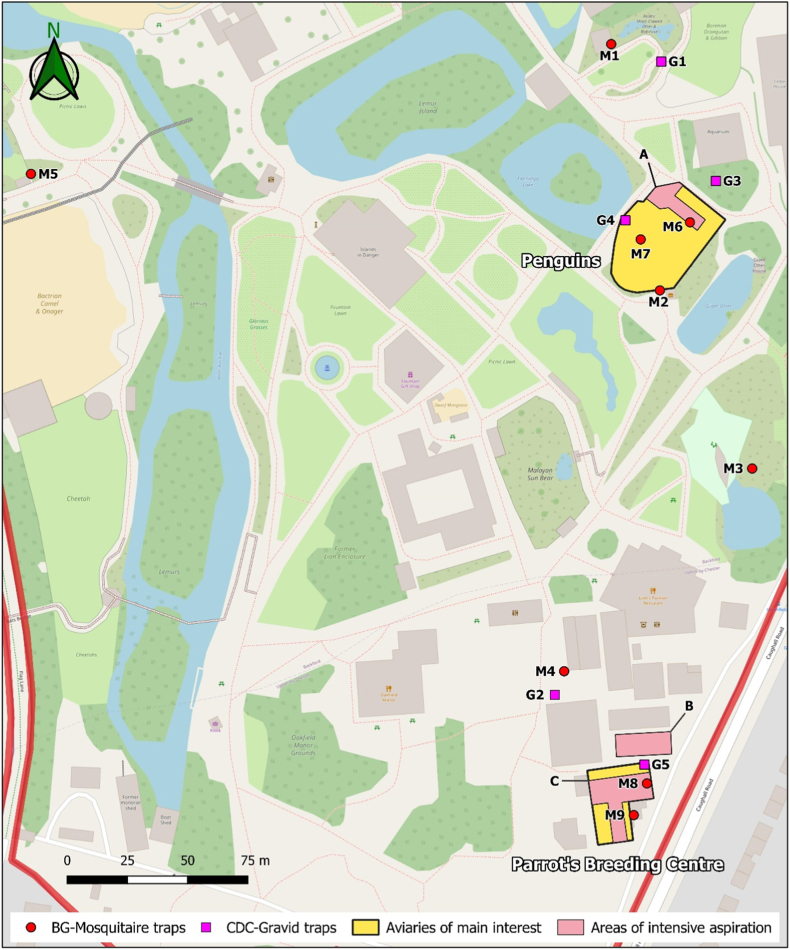
Sampling sites in Chester Zoo (Cheshire, UK). The sampling focused around two aviaries: Penguins and the Parrot breeding centre. M: BG-Mosquitaire traps; G: CDC-Gravid traps; Aspiration areas: A: area behind the Penguin's kitchen; B: equipment's shed of the botanical team; C: accessible areas inside the Parrot breeding centre.

Mosquitoes were collected in Chester Zoo (Cheshire, United Kingdom) from the 1st of September to the 2nd of October in 2020 using two traps, the Biogents-Mosquitaire (hereafter coded as M trap) and Centres for Disease Control and Prevention (CDC) Gravid trap model 1712 (hereafter coded as G trap). The M trap attracts host-seeking female, and the G trap targets gravid *Culex* mosquitoes using an oviposition attractant [25]. Both traps have proved to be effective for collecting *Culex* spp. in previous samplings at the zoo [26]. In total, nine M traps and five G traps were used, placed at least 10 m apart to minimise interference among them (Table 1, Fig. 1).

**Table 1:** Trap ID, location, and description of the sampling sites.

TRAPS	LOCATION	ANIMAL EXHIBITS^A^	OTHER CLOSE AREAS^A^	VEGETATION DENSITY AROUND THE TRAP^B^	EXPOSURE^C^	MOSQUITO DEVELOPMENT SITES^A^
M1	Garden area	Babirusa and flamingos	Small garden area and public footpaths	*Bushes:* none (ground covered with dead leaves and branches) *Trees:* dense (conifers)	None	None observed
M2	Garden area	Penguins and giant otters	Public footpaths and a snacks shop	*Bushes:* sparse (some ornamental bushes) *Trees:* none	Partial (some cover from the exhibit wall, plants and fence)	None observed
M3	Staff area	Off-show aviaries	Children's playground and picnic area	*Bushes:* medium (few bushes and weeds) *Trees:* dense (conifers)	None	Pond with limited management and reeds on one of its sides
M4	Staff area	Owls	Green houses and public footpath	*Bushes:* medium (few bushes from the fence and temporal plants under the gardener's care. *Trees:* none	Partial (some cover from the bushes and the wall of the owl's exhibit)	Water beds for high-wetness plants and water containers
M5	Garden area	Andean condor (including other bird species)	Picnic area and footpaths	*Bushes:* none *Trees:* dense (conifers)	None	None observed
M6	Staff area	Penguins	Staff area	*Bushes:* dense (several garden bushes) *Trees:* medium (some ornamental trees)	None	None observed
M7	Inside the exhibit	Penguins	Public footpath and staff area	*Bushes:* none *Trees:* none	Exposed (very limited cover from the exhibit fence)	None observed
M8	Inside an unused enclosure	Parrot breeding centre	Staff area and workshop	*Bushes:* sparse (some plants of the enclosure) *Trees:* none	None	Several plant pots with rainwater
M9	Staff area	Parrot breeding centre	Staff area and workshop	*Bushes:* none *Trees:* none	Exposed (very limited cover from the centre's wall)	Several plant pots with rainwater
G1	Garden area	None	Small garden area and public footpaths	*Bushes:* medium (ornamental bushes) *Trees:* medium (ornamental trees)	None	None observed
G2	Staff area	None	Green houses and public footpath	*Bushes:* medium (few bushes from the fence and temporal plants under the gardener's care. *Trees:* none	Partial (some cover from the bushes)	Water beds for high-wetness plants and water containers
G3	Garden area	Penguins and giant otters	Small garden and public footpath	*Bushes:* sparse (some weeds) *Trees:* sparse (some short trees)	Partial (some cover from the vegetation and the exhibits fence)	None observed
G4	Inside the exhibit	Penguins and flamingos	Public footpath and staff area	*Bushes:* none *Trees:* none	Exposed (very limited cover from the exhibit fence)	None observed
G5	Inside an unused enclosure	Parrot breeding centre	Staff area and workshop	*Bushes:* sparse (some plants of the enclosure) *Trees:* none	None	Several plant pots with rainwater

M: BG-Mosquitaire traps; G: CDC-Gravid Traps; ^a^: Within 20 m ^b^: Within 10 m, categorised as none, sparse, medium, or dense at the bush level (<2 m) and tree level (>2 m); ^c^: Exposure to direct sunlight, rain, and wind, categorised as none, partial or exposed.

Additionally, aspiration of resting mosquitoes, using a Modified CDC-backpack aspirator model 1412, was performed on the 1st, 4th^,^ and 11th of September for 5 min in the surroundings of each trap. In addition, an exhaustive aspiration, covering all accessible surfaces, was performed on the 26th of October in the area behind the penguin's kitchen, inside the equipment shed of the botanical team, and in the Parrot breeding centre (excluding enclosures with animals to avoid stressing them and offices that were not in use). Water containers around the sampling sites were inspected for larvae and pupae. Adult mosquitoes were killed by freezing at −20 °C and identified morphologically over an icepack [27]. Mosquitoes were pooled by collection date, sampling site, collection method, species, sex, fed status (unfed or blood fed) and integrity (damaged or intact). No more than 50 mosquitoes were pooled together at any time and pools were stored in 2 ml reaction tubes at −80 °C until viral testing.

### 2.2 Viral testing of mosquitoes

All pools of undamaged and unfed *Culex* spp. females were homogenised in one mL of PBS supplemented with FBS, Penicillin Streptomycin and Amphotericin B solution (Thermo Fisher Scientific, Waltham, MA, USA), using two stainless steel beads and a TissueLyser II (Qiagen, Hilden, Germany) for 2 min at 25 Hz. After homogenisation, samples were centrifuged at 5000×*g* for 2 min; 50 μl of the supernatant from each pool was then pooled with seven other samples for RNA extraction using the QIAamp Viral RNA Mini Kit (Qiagen, Hilden, Germany) according to the manufacturer's recommendations. This strategy was used to prevent compromising the possibility to trace any positive pool of extracted RNA back to its original field pool [28]. Extracted RNA was analysed with RT-qPCR protocols specific for WNV [29], USUV [30] and SINV [31]. PCR was performed using the QuantiTect Probe PCR Kit (Qiagen, Hilden, Germany) and 25 μl reaction volume with 5 μl of RNA input. All runs were performed in duplicates and positive controls of extracted WNV, USUV and SINV were used, as well as a virus-negative mosquito control. Pools of damaged mosquitoes were identified as far as possible but not tested for viruses.

### 2.3 Wild bird collections and post-mortem examination

Wild birds found dead at Chester Zoo between May and December 2020 were collected and stored at −20 °C. During post-mortem examination, a body condition score was assigned to the birds on a scale from one (emaciation) to five (overweight). Kidney and brain samples were collected and stored at −80 °C; the surfaces and equipment were sterilised between individuals using Anigene™ (Byotrol).

### 2.4 Viral testing of wild birds

Kidney and brain samples from the same individuals were pooled for RNA extraction using TRIzol™ (Thermo Fisher Scientific) following the manufacturer's instructions. Homogenisation was conducted using a disposable pestle. Real time PCR for WNV and USUV was performed using primers and probes by Lanciotti et al. [32] and Jöst et al. [30], respectively. RT-qPCRs were conducted using the Quantitect probe RT-PCR kit (Qiagen) with a final reaction volume of 10 μl. Each reaction contained 5 μl of master mix, 0.1 μl reverse transcription mix, 0.4 μl of forward and reverse primers, 0.2 μl of probe, 1.9 μl of water and 2 μl of RNA template. USUV ENT MP nucleic acid and WNV BA 80900–4 (44) (BEI Resources) were used as positive controls and nuclease-free water was used as a negative control. All reactions were run in duplicate.

### 2.5 Data analysis

Mosquito abundance was compared by sampling site, collection date, and trap type. Generalised linear models with a negative binomial family were constructed to explore temporal and spatial differences by sampling site and date. The minimum sample size needed to conclude the presence of viral infection was estimated for wild birds and mosquitoes following the formula for infinite populations given by Dohoo et al. (2003) [33]:

n = ln *α*/ln *q*

where: n = required sample size, α = 0.1, 0.05 or 0.01 for 90%, 95% and 99% confidence, respectively, q = 1 – (expected prevalence).

The expected prevalence in wild birds and *Culex* spp. mosquitoes for WNV, USUV and SINV was taken from previous European studies (Supplementary Tables S1, S2 and S3). The sample size calculations were done by host group using two values as the expected prevalence to obtain *q*, the lowest prevalence reported overall and the mean of the minimum prevalence. The latter was calculated averaging the minimum prevalence per study (some studies reported a range of prevalence or divided temporally or spatially) from all studies. Prevalence of 0% was excluded in both cases. In bird samplings, prevalence resulting from molecular detection techniques were preferred for concordance with our methods. In this way, we found that the minimum infection rate per 1000 mosquitoes was on average 1.7 for WNV, 3.1 for USUV and 10.7 for SINV, and the overall lowest rate was 0.3, 0.2 and 1.8, respectively. For bird studies, the average minimum prevalence, and the lowest prevalence were respectively 3.3% and 0.6% for WNV, 14% and 0.6% for USUV, and 9.4% and 0.3%, for SINV.

## 3 Results

### 3.1 Mosquito collections and viral testing

In total, 3316 mosquitoes were collected and sorted into 349 pools. The vast majority of these were identified as *Culex* spp. (n = 3244, 97.82%), followed by *Culiseta annulata* (n = 68, 2.05%); *Anopheles maculipennis* s.l. (n = 3, 0.09%) and *An. plumbeus* (n = 1, 0.03%). Females accounted for 95.1% of the whole collection and *Culex* spp. females represented 93.5% (n = 3100) of the mosquitoes collected. *Culex* mosquitoes sampled at the same site before, over the whole mosquito season, were identified to species using a molecular assay to reliably separate the morphologically identical females of *Cx. pipiens* s.l. from *Cx. torrentium*, showing that 99.1% of them were *Cx. pipiens* s.l. and only 0.9% *Cx. torrentium* [34]*,* the last of which was present from May to August with the highest abundance in June (data not shown).

**Fig. 2:**
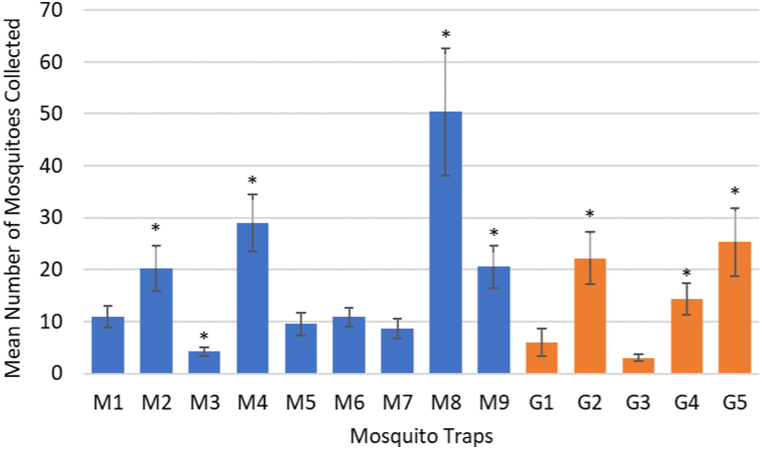
Mean number of mosquitoes captured by trap during 2020 in Chester Zoo. M: BG-Mosquitaire traps; G: CDC-Gravid traps; Error bars: standard error of the mean; Asterisks: significant differences by trap type.

**Fig. 3:**
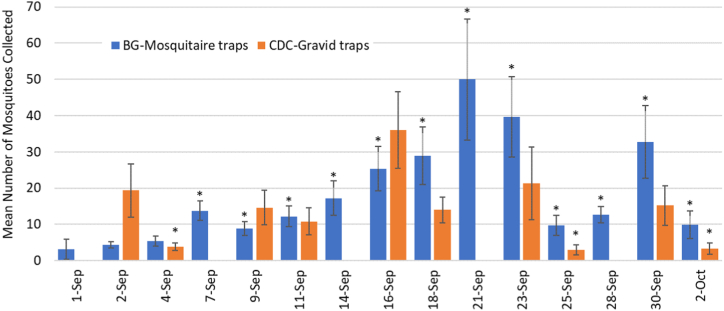
Mean number of mosquitoes captured with traps by sampling day during 2020 in Chester Zoo. CDC-Gravid traps were not operated in all dates. Error bars: standard error of the mean; Asterisks: significant differences by trap type.

The five G traps were emptied on 12 occasions and collected a total of 708 mosquitoes, the nine M traps were emptied on 15 occasions and collected 2467 mosquitoes, while the four aspiration sessions collected 141 mosquitoes. The GLM showed that the G traps G2, G4 and G5 captured significantly more mosquitoes than the other G traps (*p* < 0.001, *p* = 0.024 and *p* < 0.001, respectively) (Fig. 2). Significantly fewer mosquitoes were captured by the G traps on the 4th and 25th of September and the 2nd of October than all other dates (all *p* < 0.002) (Fig. 3). The M traps that collected more mosquitoes were M2 (*p* = 0.043), M4 (*p* = 0.001), M8 (*p* < 0.001) and M9 (*p* = 0.039) and fewer mosquitoes in area M3 (*p* = 0.003) (Fig. 2). The analysis by date showed that almost all the collections of the M traps were different from each other (all *p* < 0.013), except for the ones on the 2nd and 4th of September (Fig. 3). Through aspiration, mosquitoes were caught only in September, with 11 mosquitoes on the 1st, 13 on the 4th and 13 on the 11th. For the rest of the regular aspiration dates no mosquitoes were collected; however, a substantial number of mosquitoes were caught during the intense aspiration (26th of October) inside the shed behind the penguin kitchen (n = 33), in the equipment shed of the botanical team (n = 34) and in the Parrot breeding centre (n = 37) (Fig. S1). Twenty blood-fed mosquitoes were collected of the species *Culiseta annulata* (n = 15) and *Culex* spp. (n = 5). One *Cs. annulata* was captured by aspiration and the rest in M traps; one *Culex* spp. was captured by aspiration, another in a M trap and three in G traps.

Larvae and pupae were not observed in most sampling areas, including the water beds for plants near the greenhouses (near traps G2 and M4) where they had been abundant in previous collections [26] and were only found in three plant pots and a plastic cover containing rainwater in front of the Parrot breeding centre (Fig. S2).

Undamaged and unfed *Culex* spp. females (n = 2878) were homogenised in 192 pools, which were further pooled and used for RNA extraction in 24 pools (blood-fed mosquitoes were processed individually). None of these samples tested positive for WNV, USUV or SINV in virus specific RT-qPCRs. We collected more than enough mosquitoes to ascertain the absence of viral infection with up to 99% confidence for the three viruses when the average minimum infection rate was used to estimate the sampling size. However, when using the overall lowest infection rate for sample estimations, we collected enough mosquitoes to prove the absence of viral infection only for SINV with a confidence of 90% (Table 2).

**Table 2:** Reported prevalence of WNV, USUV and SINV in European studies, and the minimum sampling sizes required to detect the presence of viral infections with different percentages of confidence.

Mosquitoes (*Culex* spp.)					
Virus	Prevalence (base 1)		Minimum sampling size		
			90%	95%	99%
**WNV**	Min	0.0003	7674	9984	15348
	Ave	0.0017	1353	1761	2707
**USUV**	Min	0.0002	11512	14977	23024
	Ave	0.0031	742	965	1483
**SINV**	Min	0.0018	1278	1663	2556
	Ave	0.0107	214	278	428
**Wild birds**					
**Virus**	**Prevalence (base 1)**		**Minimum sampling size**		
			**90%**	**95%**	**99%**
**WNV**^**†**^	Min	0.006	383	498	765
	Ave	0.033	69	89	137
**USUV**^**†**^	Min	0.006	383	498	765
	Ave	0.14	15	20	31
**SINV**^**‡**^	Min	0.003	766	997	1533
	Ave	0.094	23	30	47

Min: minimum prevalence reported (excluding 0%); Ave: average of the minimum prevalence reported (excluding 0%). Reference prevalence was obtained from studies in Supplementary Table S1 (mosquitoes) and S2 and S3 (birds). ^†:^ Molecular testing; ^‡:^ Serological testing.

### 3.2 Investigation of dead wild birds

In total, 18 wild birds were found dead on site, comprising six different orders and 11 species, with the most abundant being Passeriformes and the Eurasian magpie (*Pica pica*) (n = 4). The birds are mostly resident species and three are of conservation concern according to the Royal Society for the Protection of Birds (RSPB) classification for UK birds [35]. Macroscopic findings are summarised in Table 3 and include splenomegaly (n = 6) and hepatomegaly (n = 1). None of the samples collected were positive for WNV or USUV by qRT-PCR, and the number of birds tested was lower than the number needed to detect viral infections for WNV and USUV except for USUV with the average of the minimum prevalence and 90% confidence (n = 15) (Table 2).

**Table 3:** Species, individual features, and post-mortem examination findings of wild birds found dead in Chester Zoo during 2020.

Bird	Order	Migratory Status	Conservation Status^a^	Sex	BCS	Age	Suspected cause of death	Description of organomegaly
Black-headed Gull	Charadriiformes	M	LC, Amber	F	2	A	Unknown	None identified
*Larus ridibundus*				M	2	A	Foreign body with perforation of GIT	Severe splenomegaly
Common Moorhen	Gruiformes	R	LC, Amber	M	3	J	Trauma	None identified
*Gallinula chloropus*				F	2	A	Unknown	None identified
Common Starling	Passeriformes	M and R	LC, Red	F	2	J	Unknown	Moderate splenomegaly
*Sturnus vulgaris*								
Common Woodpigeon	Columbiformes	R	LC, Amber	Un	3	A	Trauma	Mild splenomegaly
*Columba palumbus*				F	2	A	Unknown	None identified
Eurasian Blackbird	Passeriformes	M and R	LC, Green	F	3	J	Trauma	Moderate splenomegaly
*Turdus merula*								Mild hepatomegaly
Eurasian Collared-dove	Columbiformes	R	LC, Green	F	3	A	Unknown	None identified
*Streptopelia decaocto*								
Eurasian Green Woodpecker	Piciformes	R	LC, Green	M	3	A	Trauma	None identified
*Picus viridis*								
Eurasian Magpie	Passeriformes	R	LC, Green	F	3	A	Trauma	Mild splenomegaly
*Pica pica* Grey Heron				M	2	A	Unknown	None identified
				M	3	A	GIT pathology	None identified
				Un	Un	A	Unknown	None identified
	Pelecaniformes	M and R	LC, Green	M	2	A	Respiratory pathology	None identified
*Ardea cinerea*								
House Sparrow	Passeriformes	R	LC, Red	M	3	A	Unknown	None identified
*Passer domesticus*				M	2	A	Trauma	None identified
Mistle Thrush	Passeriformes	R	LC, Red	F	3	A	Trauma	Severe splenomegaly
*Turdus viscivorus*								

a According to the BirdLife International [50] and RSPB [35] classifications; BCS: body condition score from 1 (severe emaciation) to 5 (severe overweight); F: female; M: male; Un: unknown; A: adult; J: juvenile; M: migratory; R: resident; LC: least concern; GIT: gastrointestinal tract.

## 4 Discussion

Mosquito-borne viruses capable of infecting wild birds, domestic animals and humans have expanded their range and are now endemic in new regions of Europe, including two flaviviruses (WNV and USUV) and one alphavirus (SINV) [36,37]. Here, we undertook an integrated sampling of mosquitoes and wild birds in a zoo in the UK to investigate the prevalence of the above-mentioned arboviruses. We obtained relevant information about vectors and hosts that can guide future surveillance efforts and mosquito control strategies, despite the absence of virus evidence at this time in the zoo.

The number of mosquitoes collected (n = 3316) was unexpectedly high in relation to collections made in 2017–2019 on the same site, considering the sampling period and number of active traps [26]. Mosquito abundance was also high compared with another mosquito study in zoos, although the sampling protocol was different [11]. It is likely that environmental conditions have affected mosquito abundance as temperature and rainfall are strong drivers of mosquito populations [38]. The minimum temperature, maximum temperature and rainfall averages of 2020 were significantly higher compared to the ones from the previous twenty years (closest Met Office weather station, Shawbury, circa 50 km [39]) and it is likely that they favoured mosquito development and activity.

Differences were also observed between sampling occasions and traps despite the relatively small study area and short sampling period. Temperature in the proximity to mosquito traps was not measured but could help understanding the observed variability in mosquito abundance between sampling points. It is also possible that the concentration of zoo animals and visitors influence mosquito distribution. Despite *Cx pipiens* s.l. typically displaying ornithophilic feeding preferences, it has been found feeding on humans at this site, even with mixed human/bird bloodmeals [40]. This could be the reason for the high catches in traps G4 and M2, which are traps close to visitor footpaths and the penguins’ exhibit, which is particularly popular at this zoo. Water bodies may also attract mosquitoes and increase the number collected near them. We found abundant immature mosquitoes near the Parrot breeding centre, and all the traps nearby had high catches, with the highest ones per trap type being G5 and M8 also in this area.

*Culex* spp., an important vector for the three zoonotic arboviruses investigated here, as well as avian malaria, was the dominant species at the zoo, comprising 94% of the collected mosquitoes. Mosquitoes look for resting sites that offer protection from wind, rain, freezing temperatures, and direct sunlight. In addition, *Culex* mosquitoes overwinter as adults, and need similar protection from the environment. Overwintering mosquitoes could have a major role in the maintenance of arboviruses as up to 70% of *Cx. pipiens* s.l. may survive over four months under optimal conditions, without detrimental effects on vector competence for WNV [41] and could present an infection rate of 4.6% for SINV in endemic regions [42]. Distinguishing resting from overwintering mosquitoes with certainty requires the assessment of their physiological status through ovary dissection [42]. As our sampling concluded towards the end of the mosquito season, the aspirated specimens could represent either of these cases; nevertheless, the aspiration sampling allowed us to identify potential resting or overwintering sites in proximity to aviaries.

Wild and captive birds are regularly present in zoo grounds and thus act as natural sentinels for pathogen detection, which makes them highly suitable for surveillance purposes [15,43]. Viral testing of wild birds showed that none were infected with WNV or USUV, and most likely died from severe traumatic injuries and not from viral infection. Hepatomegaly and splenomegaly have been observed in USUV and WNV infections, but they are considered non-specific and could also be observed in relation to other infectious (e.g., *Plasmodium* spp.) and non-infectious pathogenic agents [44,45]. Pathological findings are not always reported in arbovirus investigations in wild birds, and macroscopic lesions are non-specific and thus of limited diagnostic value on their own; however, this information would be valuable and obtainable for situations in which birds can be methodically examined, such as in zoos and rehabilitation centres.

Estimations of the minimum sampling size needed to establish the absence of arboviruses in mosquitoes and birds is a helpful guideline, but several considerations are needed. Most importantly, sample size estimations are based on a previous reported prevalence that depends on surveillance objectives and thus are influenced by geographic, environmental, temporal, ecological, and methodological factors. Here, we conservatively estimated the sample size using the lowest prevalence reported overall and the average of the minimum prevalence per study to partially compensate for these biases. Estimations with the highest confidence based on the overall lowest prevalence result in sample sizes are hard to achieve in regular surveillance and therefore, average minimum prevalence could be a more achievable guideline. With our sample size, we achieved the statistical power to demonstrate the absence of the three viral infections with a confidence of 99%, based on the average of the lowest minimum infection rate. Therefore, the probability of these viruses being present in the mosquito population at the time of the sampling is minimal. Nonetheless, we did not have enough bird samples to confirm the absence of infection, except for USUV (calculated with the average of the minimum prevalence with a 90% confidence), but this estimation could have been biased due to the high prevalence reported in endemic regions.

The introduction of exotic pathogens through natural routes, such as bird migration, may be uncommon, but it can lead to viral establishment and recurrence of endemic outbreaks, as in the case of SINV introduction and establishment in northern Europe [36]. In the UK, neutralizing antibodies to WNV, USUV and SINV have been reported in wild resident birds, suggesting that these viruses have circulated within the country [46], although no further evidence was presented until 2020, when USUV was isolated from wild birds in London confirming virus circulation [4]. Zoological gardens are advantageous locations for the surveillance of exotic pathogens due to the high diversity of species representing a wide susceptibility range, close medical monitoring, access to archived samples and clinical information, availability of diagnostic skills and tools, and institutional collaborations [7,47]. Moreover, zoos can also provide information for the conservation of local species by monitoring their populations. Three species of wild birds sampled in this study are in the RSPB Red List, and additional stress, such as infectious diseases, could adversely affect their long-term survival. Therefore, zoos should be incorporated in surveillance efforts, especially of emerging or exotic pathogens.

Previous studies in Chester Zoo have found *Plasmodium* spp. parasites circulating in mosquitoes and birds at Chester Zoo [34] and mosquitoes feeding in humans and birds [40], which demonstrates an active transmission cycle involving *Culex* spp. vectors and bird reservoirs that could represent a health risk to susceptible animals and humans if zoonotic arboviruses become established. As *Culex* spp. is a relevant vector of WNV, SINV, USUV, *Plasmodium* and filarial nematodes [7,11], the regular surveillance and control of this mosquito should be continued in Chester Zoo, including the management of water sources, monitoring of adult mosquitoes, removal of resting or overwintering mosquitoes, xenomonitoring, and assessment of mosquito control measures. In some surveys, mosquitoes have tested positive for flavivirus while birds have been negative [48], and vice versa [49]; therefore, concurrent monitoring of both hosts, vectors, and birds, is recommended.

## 5 Conclusions

Relevant data regarding mosquito abundance, distribution, developing and resting sites, as well as the bird community, were collected in this study; therefore, we consider that integrated surveillance of pathogens in zoos is highly advantageous for the detection of exotic pathogens and monitoring of endemic ones. We found no evidence that WNV, USUV or SINV circulated within the mosquito population at Chester Zoo in September 2020 with a high level of confidence in our sample size estimations. Nonetheless, the high abundance of *Culex* spp. in the proximities of bird enclosures motivate continued mosquito monitoring and implementing efficient control actions like the regular cleaning of water containers and removing resting or overwintering mosquitoes in relevant locations. Integrated surveillance of zoonotic arboviruses in wild birds and mosquitoes, including immature, host-seeking, gravid and resting or overwintering mosquitoes in zoos could promptly identify pathogen introductions and inform about potential exposure and transmission risks for susceptible animals and people.

## Funding

This research was financially supported by 10.13039/501100005113North of England Zoological Society (10.13039/501100005359Chester Zoo). This work was additionally funded by the 10.13039/501100000272National Institute for Health Research (10.13039/501100000272NIHR) Health Protection Research Unit in Emerging and Zoonotic Infections at the 10.13039/501100000836University of Liverpool, in partnership with the 10.13039/100007397UK Health Security Agency, the 10.13039/100014976Liverpool School of Tropical Medicine and the 10.13039/501100000769University of Oxford. NS and MB are based at University of Liverpool. The views expressed are those of the authors and not necessarily the ones of the NHS, the NIHR, the Department of Health and Social Care or the UKHSA. The 10.13039/501100009252SciLifeLab Pandemic Preparedness projects (LPP1-007 and REPLP1:005) also contribute to the funding of this project. The funding sources were not involved with the methodology or conclusions of this paper.

## Ethic declaration

Ethical approval for the work carried out in this study was obtained from the Chester Zoo science committee and University of Liverpool Veterinary Research Ethics Committee (reference VREC532a).

## Consent for publication

Not applicable.

## Data availability

Data from this study has not been deposited in any repository as most of it is presented in the main text and supplementary material. However, additional data are available from the corresponding author upon reasonable request.

Manuscript: Surveillance of Culex spp. vectors and zoonotic arboviruses at a zoo in the United Kingdom, by Hernandez-Colina et al.

## CRediT authorship contribution statement

**Arturo Hernandez-Colina:** Writing – review & editing, Writing – original draft, Visualization, Validation, Project administration, Methodology, Investigation, Funding acquisition, Formal analysis, Data curation, Conceptualization. **Nicola Seechurn:** Writing – review & editing, Visualization, Validation, Methodology, Investigation. **Taiana Costa:** Writing – review & editing, Validation, Methodology, Investigation. **Javier Lopez:** Writing – review & editing, Supervision, Resources, Project administration, Methodology, Funding acquisition, Conceptualization. **Matthew Baylis:** Writing – review & editing, Validation, Supervision, Resources, Project administration, Methodology, Investigation, Funding acquisition, Conceptualization. **Jenny C. Hesson:** Writing – review & editing, Validation, Supervision, Resources, Project administration, Methodology, Investigation, Funding acquisition, Conceptualization.

## Declaration of competing interest

The authors declare that they have no known competing financial interests or personal relationships that could have appeared to influence the work reported in this paper.
