# Supplementary material for: Surveillance of Culex spp. vectors and zoonotic arboviruses at a zoo in the United Kingdom
Source: Heliyon. 2024 Feb 15;10(4):e26477. doi: 10.1016/j.heliyon.2024.e26477 (PMC10884501; doi:10.1016/j.heliyon.2024.e26477)
Supplement: Multimedia component 1 [file mmc1.pdf]

Supplementary Tables

Table S1. Reported minimum infection rate (over 1000 mosquitoes) of bird related arboviruses in *Culex* spp. in Europe.

Mosquito species	West Nile virus	Usutu virus	Sindbis virus	Country	Reference
Culex spp.	6.8	6.8	NT	Austria	Bakran-Lebl et al. (2022)
Culex spp.	0.5	NT	NT	Cyprus	Pallari et al. (2022)
Culex spp.	0	0.7 - 13.7	NT	Croatia	Klobucar et al. (2021)
Culex spp.	NT	NT	45.6[b]	Sweden	Bergman et al. (2020)
Culex spp.	4.6	0	NT	Netherlands	Sikkema et al. (2020)
Culex spp.	4 - 12.5	5.8	NT	Slovakia	Cabanova et al. (2019)
Cx. p. pipiens / Culex spp.	NT	44.4 - 54.1[a]	NT	Austria	Camp et al. (2019)
Culex spp.	NT	5.7	NT	Italy	Carletti et al. (2019)
Culex spp.	0	0	0	Sweden	Hesson et al. (2019)
Culex spp. [z]	0	0	2.4	Germany	Heym et al. (2019)
Culex spp.	NT	NT	4.7 – 10.3	Sweden	Lundstrom et al. (2019)
Culex spp.	0	0.7	NT	Croatia	Vilibic-Cavlek et al. (2019)
Culex spp.	NT	0.3	NT	Serbia	Kemenesi et al. (2018)
Culex spp.	0.5 – 7.4	NT	NT	Austria	Kolodziejek et al. (2018)
Culex pipiens	4.8	NT	NT	Greece	Mavridis et al. (2018)
Culex spp.	0.4 – 0.5*	NT	NT	Serbia	Petrović et al. (2018)
Culex spp.	3.3	4.4	NT	Italy	Mancini et al. (2017)
Culex spp.	0.3	NT	NT	Serbia	Petric et al. (2017)
Culex spp.	0.3 – 15	NT	NT	Greece	Patsoula et al. (2016)
Culex spp.	0.4	0.5	NT	Italy	Calzolari et al. (2015)
Cx. torrentium<br>*Cx. pipiens*	NT<br>NT	NT<br>NT	36.5<br>8.2	Sweden	Hesson et al. (2015)
Culex perexiguus	1.2	0.2	NT	Spain	Vázquez et al. (2011)
Culex spp.	NT	6	NT	Spain	Busquets et al. (2008)
**Minimum‡**	**0.3**	**0.2**	**1.8**		
**Average of min.‡**	**1.7**	**3.1**	**10.7**		

[z]: mosquitoes collected in zoos; ‡: excluding 0% prevalence; NT: not tested; *: estimated from the number of pools and the maximum pool size as the total of mosquitoes caught is not given; [a]: targeted sampling in an area with confirmed dead blackbirds linked to USUV (excluded from sample size calculations); [b]: overwintering mosquitoes (excluded from sample size calculations).

Table S2. Reported prevalence in percentage by RT-qPCR testing of bird related arboviruses in wild and captive birds in Europe.

Bird origin	West Nile virus	Usutu virus	Country	Reference
wild / zoo	NT	0.6	United Kingdom	Folly et al. (2022)
wild	NT	5.2	United Kingdom	Lawson et al. (2022)
wild / zoo	0.7 - 0.9	1.2 - 12.2	Germany	Ziegler et al. (2022)
wild	NT	27.3	Belgium	Benzarti et al. (2020)
wild	0.6	0	The Netherlands	Sikkema et al. (2020)
wild	NT	17 - 36.4	Czech Republic	Honig et al. (2019)
wild	0	3.2 - 3.9	Germany	Michel et al. (2019)
wild	5.7	2.9	Croatia	Vilibic-Cavlek et al. (2019)
wild	1.1 - 1.5	NT	Serbia	Petrović et al. (2018)
wild	NT	28.6	Austria	Bakonyi et al. (2017)
wild	NT	21.7	Hungary	
wild	1.8 - 11	NT	Serbia	Petric et al. (2017)
wild	NT	20	Spain	Hofle et al. (2013)
wild	10	NT	Serbia	Petrović et al. (2013)
wild / zoo	NT	38.6	Germany	Becker et al. (2012)
wild / zoo	0	NT	Portugal	Barros et al. (2011)
wild	NT	1.1	Italy	Savini et al. (2011)
**Minimum‡**	**0.6**	**0.6**		
**Average of min.‡**	**3.3**	**14**		

‡: excluding 0% prevalence; NT: not tested. Results were summarised by sampling year and region (as given by the authors) for estimating the prevalence range. Targeted testing of suspected infected birds was not considered for sample size calculations.

Table S3. Reported prevalence in percentage of bird related arbovirus antibodies in wild and captive birds in Europe.

Bird origin	West Nile virus	Usutu virus	Sindbis virus	Country	Reference
wild	8.7	2.7	0	Romania	Coroian et al. (2022)
wild / zoo	NT	11.6	NT	United Kingdom	Folly et al. (2022)
wild	19.2	1	NT	Spain	Marzal et al. (2022)
zoo	6.5 -6.6	10.5 - 46.7	NT	Germany	Santos et al. (2022)
wild / zoo	14.8 - 16.2	3.11 - 7.2	NT	Germany	Ziegler et al. (2022)
wild	18.2	1	NT	Spain	Bravo-Barriga et al. (2021)
zoo	2.9	2.2	NT	Slovenia	Kvapil et al. (2021)
zoo	1.5	14.6	NT	France	Constant et al. (2020)
wild	0.6	0	NT	The Netherlands	Sikkema et al. (2020)
wild	2.3 - 6.5	1.8 - 21.1	NT	Germany	Michel et al. (2019)
wild	3.4 - 17.1	NT	NT	Spain	Napp et al. (2019)
wild	8.8	NT	NT	Romania	Vasic' et al. (2019)
wild	NT	NT	1.2 - 12.3	Germany	Ziegler et al. (2019)
wild	1.3	0.7	NT	Spain	Ferraguti et al. (2016)
wild	NT	NT	2.5 - 43.6	Sweden	Hesson et al. (2016)
wild	1.1	3.6	NT	Spain	Jurado-Tarifa et al. (2016)
wild	2.1	2.1	NT	Spain	Cano-Terriza et al. (2015)
zoo	2	2	NT	Spain	
zoo	NT	8.6	NT	Austria	Buchebner et al. (2013)
zoo	NT	5.3	NT	Switzerland	
zoo	NT	6.6	NT	Switzerland	
zoo	NT	0	NT	Hungary	
wild	23	10	NT	Spain	Llorente et al. (2013)
wild	5	NT	NT	Serbia	Petrović et al. (2013)
wild / zoo	19.8	NT	NT	Portugal	Barros et al. (2011)
wild	NT	8.2	NT	Italy	Savini et al. (2011)
wild ^{r}	NT	NT	1.4 - 27.4	Finland	Kurkela et al. (2008)
wild ^{m}	NT	NT	0.3 - 1		
wild	14.7	53.8	50.4	United Kingdom	Buckley et al. (2003)
wild	NT	NT	2 - 12.1	Sweden	Lundstrom et al. (2001)
wild	NT	NT	8	Sweden	Lundstrom et al. (1992)
**Minimum‡**	**0.6**	**0.7**	**0.3**		
**Average of min.‡**	**8.2**	**7.9**	**9.4**		

‡: excluding 0% prevalence; NT: not tested; ^{r}: resident; ^{m}: migratory. Results were summarised by sampling year and region (as given by the authors) for estimating the prevalence range. Targeted testing of suspected infected birds was not considered for sample size calculations. Virus neutralisation test results were preferred over ELISA results where available.

References

Bakonyi, T., Erdelyi, K., Brunthaler, R., Dan, A., Weissenbock, H., & Nowotny, N. (2017). Usutu virus, Austria and Hungary, 2010-2016. *Emerg Microbes Infect, 6*(10), e85. doi:10.1038/emi.2017.72

Bakran-Lebl, K., Camp, J. V., Kolodziejek, J., Weidinger, P., Hufnagl, P., Cabal Rosel, A., . . . Nowotny, N. (2022). Diversity of West Nile and Usutu virus strains in mosquitoes at an international airport in Austria. *Transboundary and Emerging Diseases, 69*(4), 2096-2109. doi:10.1111/tbed.14198

Barros, S. C., Ramos, F., Fagulha, T., Duarte, M., Henriques, M., Luis, T., & Fevereiro, M. (2011). Serological evidence of West Nile virus circulation in Portugal. *Vet Microbiol, 152*(3-4), 407-410. doi:10.1016/j.vetmic.2011.05.013

Becker, N., Jost, H., Ziegler, U., Eiden, M., Hoper, D., Emmerich, P., . . . Schmidt-Chanasit, J. (2012). Epizootic emergence of Usutu virus in wild and captive birds in Germany. *PLoS One, 7*(2), e32604. doi:10.1371/journal.pone.0032604

Benzarti, E., Sarlet, M., Franssen, M., Cadar, D., Schmidt-Chanasit, J., Rivas, J. F., . . . Garigliany, M. (2020). Usutu Virus Epizootic in Belgium in 2017 and 2018: Evidence of Virus Endemization and Ongoing Introduction Events. *Vector Borne Zoonotic Dis, 20*(1), 43-50. doi:10.1089/vbz.2019.2469

Bergman, A., Dahl, E., Lundkvist, A., & Hesson, J. C. (2020). Sindbis Virus Infection in Non-Blood-Fed Hibernating Culex pipiens Mosquitoes in Sweden. *Viruses, 12*(12), 1441. doi:10.3390/v12121441

Bravo-Barriga, D., Aguilera-Sepúlveda, P., Guerrero-Carvajal, F., Llorente, F., Reina, D., Pérez-Martín, J. E., . . . Frontera, E. (2021). West Nile and Usutu virus infections in wild birds admitted to rehabilitation centres in Extremadura, western Spain, 2017–2019. *Veterinary Microbiology, 255*. doi:10.1016/j.vetmic.2021.109020

Buchebner, N., Zenker, W., Wenker, C., Steinmetz, H. W., Sos, E., Lussy, H., & Nowotny, N. (2013). Low Usutu virus seroprevalence in four zoological gardens in central Europe. *BMC veterinary research, 9*, 153. doi:10.1186/1746-6148-9-153

Buckley, A., Dawson, A., Moss, S. R., Hinsley, S. A., Bellamy, P. E., & Gould, E. A. (2003). Serological evidence of West Nile virus, Usutu virus and Sindbis virus infection of birds in the UK. *J Gen Virol, 84*(Pt 10), 2807-2817. doi:10.1099/vir.0.19341-0

Busquets, N., Alba, A., Allepuz, A., Aranda, C., & Ignacio Nunez, J. (2008). Usutu virus sequences in Culex pipiens (Diptera: Culicidae), Spain. *Emerg Infect Dis, 14*(5), 861-863. doi:10.3201/eid1405.071577

Čabanová, V., Šikutová, S., Straková, P., Šebesta, N., Vichová, B., Zubríková, D., . . . Rudolf, I. (2019). Co-circulation of west nile and usutu flaviviruses in mosquitoes in slovakia, 2018. *Viruses, 11*(7). doi:10.3390/v11070639

Calzolari, M., Pautasso, A., Montarsi, F., Albieri, A., Bellini, R., Bonilauri, P., . . . Capelli, G. (2015). West Nile virus surveillance in 2013 via mosquito screening in northern Italy and the influence of weather on virus circulation. *PLoS One, 10*(10). doi:10.1371/journal.pone.0140915

Camp, J. V., Kolodziejek, J., & Nowotny, N. (2019). Targeted surveillance reveals native and invasive mosquito species infected with Usutu virus. *Parasit Vectors, 12*(1), 46. doi:10.1186/s13071-019-3316-z

Cano-Terriza, D., Guerra, R., Lecollinet, S., Cerda-Cuellar, M., Cabezon, O., Almeria, S., & Garcia-Bocanegra, I. (2015). Epidemiological survey of zoonotic pathogens in feral pigeons (Columba livia var. domestica) and sympatric zoo species in Southern Spain. *Comp Immunol Microbiol Infect Dis, 43*, 22-27. doi:10.1016/j.cimid.2015.10.003

Carletti, F., Colavita, F., Rovida, F., Percivalle, E., Baldanti, F., Ricci, I., . . . Castilletti, C. (2019). Expanding Usutu virus circulation in Italy: detection in the Lazio region, central Italy, 2017 to 2018. *Euro Surveill, 24*(3), 1800649. doi:10.2807/1560-7917.ES.2019.24.3.1800649

Constant, O., Bollore, K., Cle, M., Barthelemy, J., Foulongne, V., Chenet, B., . . . Simonin, Y. (2020). Evidence of Exposure to USUV and WNV in Zoo Animals in France. *Pathogens, 9*(12). doi:10.3390/pathogens9121005

Coroian, M., Silaghi, C., Tews, B. A., Baltag, E. S., Marinov, M., Alexe, V., . . . Mihalca, A. D. (2022). Serological Survey of Mosquito-Borne Arboviruses in Wild Birds from Important Migratory Hotspots in Romania. *Pathogens, 11*(11). doi:10.3390/pathogens11111270

Ferraguti, M., La Puente, J. M.-D. E., Soriguer, R., Llorente, F., JimÉNez-Clavero, M. Á., & Figuerola, J. (2016). West Nile virus-neutralizing antibodies in wild birds from southern Spain. *Epidemiology and Infection, 144*(9), 1907-1911. doi:10.1017/S0950268816000133

Folly, A. J., Sewgobind, S., Hernández-Triana, L. M., Mansfield, K. L., Lean, F. Z. X., Lawson, B., . . . Johnson, N. (2022). Evidence for overwintering and autochthonous transmission of Usutu virus to wild birds following its redetection in the United Kingdom. *Transboundary and Emerging Diseases*. doi:10.1111/tbed.14738

Hesson, J. C., Lundin, E., Lundkvist, A., & Lundstrom, J. O. (2019). Surveillance of mosquito vectors in Southern Sweden for Flaviviruses and Sindbis virus. *Infect Ecol Epidemiol, 9*(1), 1698903. doi:10.1080/20008686.2019.1698903

Hesson, J. C., Lundstrom, J. O., Tok, A., Ostman, O., & Lundkvist, A. (2016). Temporal Variation in Sindbis Virus Antibody Prevalence in Bird Hosts in an Endemic Area in Sweden. *PLoS One, 11*(8), e0162005. doi:10.1371/journal.pone.0162005

Hesson, J. C., Verner-Carlsson, J., Larsson, A., Ahmed, R., Lundkvist, A., & Lundstrom, J. O. (2015). Culex torrentium Mosquito Role as Major Enzootic Vector Defined by Rate of Sindbis Virus Infection, Sweden, 2009. *Emerg Infect Dis, 21*(5), 875-878. doi:10.3201/eid2105.141577

Heym, E. C., Kampen, H., Krone, O., Schafer, M., & Werner, D. (2019). Molecular detection of vector-borne pathogens from mosquitoes collected in two zoological gardens in Germany. *Parasitol Res, 118*(7), 2097-2105. doi:10.1007/s00436-019-06327-5

Höfle, U., Gamino, V., de Mera, I. F., Mangold, A., Ortíz, J.-A., & de la Fuente, J. (2013). Usutu Virus in Migratory Song Thrushes, Spain. *Emerging Infectious Disease journal, 19*(7), 1173. doi:10.3201/eid1907.130199

Hönig, V., Palus, M., Kaspar, T., Zemanova, M., Majerova, K., Hofmannova, L., . . . Ruzek, D. (2019). Multiple lineages of usutu virus (Flaviviridae, flavivirus) in blackbirds (turdus merula) and mosquitoes (culex pipiens, cx. modestus) in the czech republic (2016–2019). *Microorganisms, 7*(11). doi:10.3390/microorganisms7110568

International, B. (2023). IUCN Red List for birds. Retrieved from http://www.birdlife.org

Jurado-Tarifa, E., Napp, S., Lecollinet, S., Arenas, A., Beck, C., Cerda-Cuellar, M., . . . Garcia-Bocanegra, I. (2016). Monitoring of West Nile virus, Usutu virus and Meaban virus in waterfowl used as decoys and wild raptors in southern Spain. *Comp Immunol Microbiol Infect Dis, 49*, 58-64. doi:10.1016/j.cimid.2016.10.001

Kemenesi, G., Buzas, D., Zana, B., Kurucz, K., Krtinic, B., Kepner, A., . . . Jakab, F. (2018). First genetic characterization of Usutu virus from Culex pipiens mosquitoes Serbia, 2014. *Infect Genet Evol, 63*, 58-61. doi:10.1016/j.meegid.2018.05.012

Klobucar, A., Savic, V., Posavec, M. C., Petrinic, S., Kuhar, U., Toplak, I., . . . Vilibic-Cavlek, T. (2021). Screening of mosquitoes for west nile virus and usutu virus in Croatia, 2015–2020. *Tropical Medicine and Infectious Disease, 6*(2). doi:10.3390/tropicalmed6020045

Kolodziejek, J., Jungbauer, C., Aberle, S. W., Allerberger, F., Bago, Z., Camp, J. V., . . . Nowotny, N. (2018). Integrated analysis of human-animal-vector surveillance: West Nile virus infections in Austria, 2015-2016. *Emerg Microbes Infect, 7*(1), 25. doi:10.1038/s41426-018-0021-5

Kurkela, S., Raetti, O., Huhtamo, E., Uzcateguli, N. Y., Nuorti, J. P., Laakkonen, J., . . . Vapalahti, O. (2008). Sindbis virus infection in resident birds, migratory birds, and humans, Finland. *Emerg Infect Dis, 14*(1), 41-47. doi:10.3201/eid1401.070510

Kvapil, P., Račnik, J., Kastelic, M., Bártová, E., Korva, M., Jelovšek, M., & Zupanc, T. (2021). A Sentinel Serological Study in Selected Zoo Animals to Assess Early Detection of West Nile and Usutu Virus Circulation in Slovenia. *Viruses, 13*, 626. doi:10.3390/v13040626

Lawson, B., Robinson, R. A., Briscoe, A. G., Cunningham, A. A., Fooks, A. R., Heaver, J. P., . . . Folly, A. J. (2022). Combining host and vector data informs emergence and potential impact of an Usutu virus outbreak in UK wild birds. *Scientific Reports, 12*(1), 10298. doi:10.1038/s41598-022-13258-2

Llorente, F., Pérez-Ramírez, E., Fernández-Pinero, J., Soriguer, R., Figuerola, J., & Jiménez-Clavero, M. Á. (2013). Flaviviruses in Game Birds, Southern Spain, 2011–2012. *Emerging Infectious Disease journal, 19*(6), 1023. doi:10.3201/eid1906.130122

Lundström, J. O., Hesson, J. C., Schäfer, M. L., Östman, Ö., Semmler, T., Bekaert, M., . . . Pfeffer, M. (2019). Sindbis virus polyarthritis outbreak signalled by virus prevalence in the mosquito vectors. *PLoS neglected tropical diseases, 13*(8), e0007702. doi:10.1371/journal.pntd.0007702

Lundstrom, J. O., Lindstrom, K. M., Olsen, B., Dufva, R., & Krakower, D. S. (2001). Prevalence of sindbis virus neutralizing antibodies among Swedish passerines indicates that thrushes are the main amplifying hosts. *J Med Entomol, 38*(2), 289-297. doi:10.1603/0022-2585-38.2.289

Lundstrom, J. O., Turell, M. J., & Niklasson, B. (1992). Antibodies to Ockelbo virus in three orders of birds (Anseriformes, Galliformes and Passeriformes) in Sweden. *J Wildl Dis, 28*(1), 144-147. doi:10.7589/0090-3558-28.1.144

Mancini, G., Montarsi, F., Calzolari, M., Capelli, G., Dottori, M., Ravagnan, S., . . . Savini, G. (2017). Mosquito species involved in the circulation of West Nile and Usutu viruses in Italy. *Vet Ital, 53*(2), 97-110. doi:10.12834/VetIt.114.933.4764.2

Marzal, A., Ferraguti, M., Muriel, J., Magallanes, S., Ortiz, J. A., Garcia-Longoria, L., . . . Frontera, E. (2022). Circulation of zoonotic flaviviruses in wild passerine birds in Western Spain. *Vet Microbiol, 268*, 109399. doi:10.1016/j.vetmic.2022.109399

Mavridis, K., Fotakis, E. A., Kioulos, I., Mpellou, S., Konstantas, S., Varela, E., . . . Vontas, J. (2018). Detection of West Nile Virus - Lineage 2 in Culex pipiens mosquitoes, associated with disease outbreak in Greece, 2017. *Acta Trop, 182*, 64-68. doi:10.1016/j.actatropica.2018.02.024

Michel, F., Sieg, M., Fischer, D., Keller, M., Eiden, M., Reuschel, M., . . . Ziegler, U. (2019). Evidence for west nile virus and usutu virus infections in wild and resident birds in Germany, 2017 and 2018. *Viruses, 11*(7). doi:10.3390/v11070674

Napp, S., Montalvo, T., Pinol-Baena, C., Gomez-Martin, M. B., Nicolas-Francisco, O., Soler, M., & Busquets, N. (2019). Usefulness of Eurasian Magpies (Pica pica) for West Nile virus Surveillance in Non-Endemic and Endemic Situations. *Viruses, 11*(8), 716. doi:10.3390/v11080716

Pallari, C. T., Christodoulou, V., Koliou, M., & Kirschel, A. N. G. (2022). First detection of WNV RNA presence in field-collected mosquitoes in Cyprus. *Acta Trop, 231*, 106470. doi:10.1016/j.actatropica.2022.106470

Patsoula, E., Vakali, A., Balatsos, G., Pervanidou, D., Beleri, S., Tegos, N., . . . Hadjichristodoulou, C. (2016). West Nile Virus Circulation in Mosquitoes in Greece (2010–2013). *BioMed research international, 2016*, 2450682. doi:10.1155/2016/2450682

Petric, D., Petrovic, T., Hrnjakovic Cvjetkovic, I., Zgomba, M., Milosevic, V., Lazic, G., . . . Petric, M. (2017). West Nile virus 'circulation' in Vojvodina, Serbia: Mosquito, bird, horse and human surveillance. *Mol Cell Probes, 31*, 28-36. doi:10.1016/j.mcp.2016.10.011

Petrovic, T., Blazquez, A. B., Lupulovic, D., Lazic, G., Escribano-Romero, E., Fabijan, D., . . . Saiz, J. (2013). Monitoring West Nile virus (WNV) infection in wild birds in Serbia during 2012: first

isolation and characterisation of WNV strains from Serbia. *Euro Surveill, 18*(44), 20622. doi:10.2807/1560-7917.es2013.18.44.20622

Petrović, T., Šekler, M., Petrić, D., Lazić, S., Debeljak, Z., Vidanović, D., . . . Plavšić, B. (2018). Methodology and results of integrated WNV surveillance programmes in Serbia. *PLoS One, 13*(4), e0195439. doi:10.1371/journal.pone.0195439

RSPB, T. R. S. f. t. P. o. B. (2023). Wildlife Guides: Birds. Retrieved from https://www.rspb.org.uk/birds-and-wildlife/wildlife-guides/bird-a-z/

Santos, P. D., Michel, F., Wylezich, C., Höper, D., Keller, M., Holicki, C. M., . . . Ziegler, U. (2022). Co-infections: Simultaneous detections of West Nile virus and Usutu virus in birds from Germany. *Transboundary and Emerging Diseases, 69*(2), 776-792. doi:10.1111/tbed.14050

Savini, G., Monaco, F., Terregino, C., Di Gennaro, A., Bano, L., Pinoni, C., . . . Lelli, R. (2011). Usutu virus in Italy: an emergence or a silent infection? *Vet Microbiol, 151*(3-4), 264-274. doi:10.1016/j.vetmic.2011.03.036

Sikkema, R. S., Schrama, M., van den Berg, T., Morren, J., Munger, E., Krol, L., . . . van der Jeugd, H. (2020). Detection of West Nile virus in a common whitethroat (Curruca communis) and Culex mosquitoes in the Netherlands, 2020. *Euro Surveill, 25*(40), 2001704. doi:10.2807/1560-7917.ES.2020.25.40.2001704

Vasić, A., Oșlobanu, L. E., Marinov, M., Crivei, L. A., Rățoi, I. A., Aniță, A., . . . Vaselek, S. (2019). Evidence of West Nile virus (WNV) circulation in wild birds and WNV RNA negativity in mosquitoes of the Danube Delta Biosphere Reserve, Romania, 2016. *Tropical Medicine and Infectious Disease, 4*(3). doi:10.3390/tropicalmed4030116

Vázquez, A., Ruiz, S., Herrero, L., Moreno, J., Molero, F., Magallanes, A., . . . Tenorio, A. (2011). West Nile and Usutu Viruses in Mosquitoes in Spain, 2008–2009. *The American Society of Tropical Medicine and Hygiene, 85*(1), 178-181. doi:10.4269/ajtmh.2011.11-0042

Vilibic-Cavlek, T., Savic, V., Sabadi, D., Peric, L., Barbic, L., Klobucar, A., . . . Savini, G. (2019). Prevalence and molecular epidemiology of West Nile and Usutu virus infections in Croatia in the 'One health' context, 2018. *Transboundary and Emerging Diseases, 66*(5), 1946-1957. doi:10.1111/tbed.13225

Ziegler, U., Bergmann, F., Fischer, D., Müller, K., Holicki, C. M., Sadeghi, B., . . . Groschup, M. H. (2022). Spread of West Nile Virus and Usutu Virus in the German Bird Population, 2019–2020. *Microorganisms, 10*(4). doi:10.3390/microorganisms10040807

Ziegler, U., Fischer, D., Eiden, M., Reuschel, M., Rinder, M., Muller, K., . . . Keller, M. (2019). Sindbis virus- a wild bird associated zoonotic arbovirus circulates in Germany. *Vet Microbiol, 239*, 108453. doi:10.1016/j.vetmic.2019.108453
