# Supplementary material for: Surveillance of Culex spp. vectors and zoonotic arboviruses at a zoo in the United Kingdom
Source: Heliyon. 2024 Feb 15;10(4):e26477. doi: 10.1016/j.heliyon.2024.e26477 (PMC10884501; doi:10.1016/j.heliyon.2024.e26477)
Supplement: Multimedia component 2 [file mmc2.pdf]

Supplementary Figures

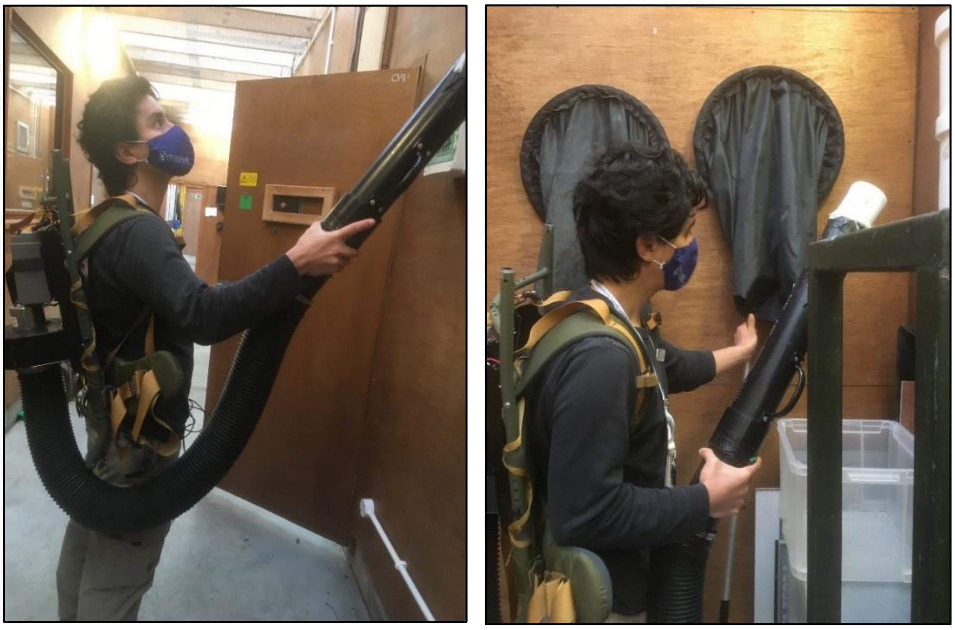

Figure S1. Aspiration of resting mosquitoes in the Parrot's Breeding Centre, Chester Zoo. Many mosquitoes were found resting behind the hand-held nets for capturing collection birds.

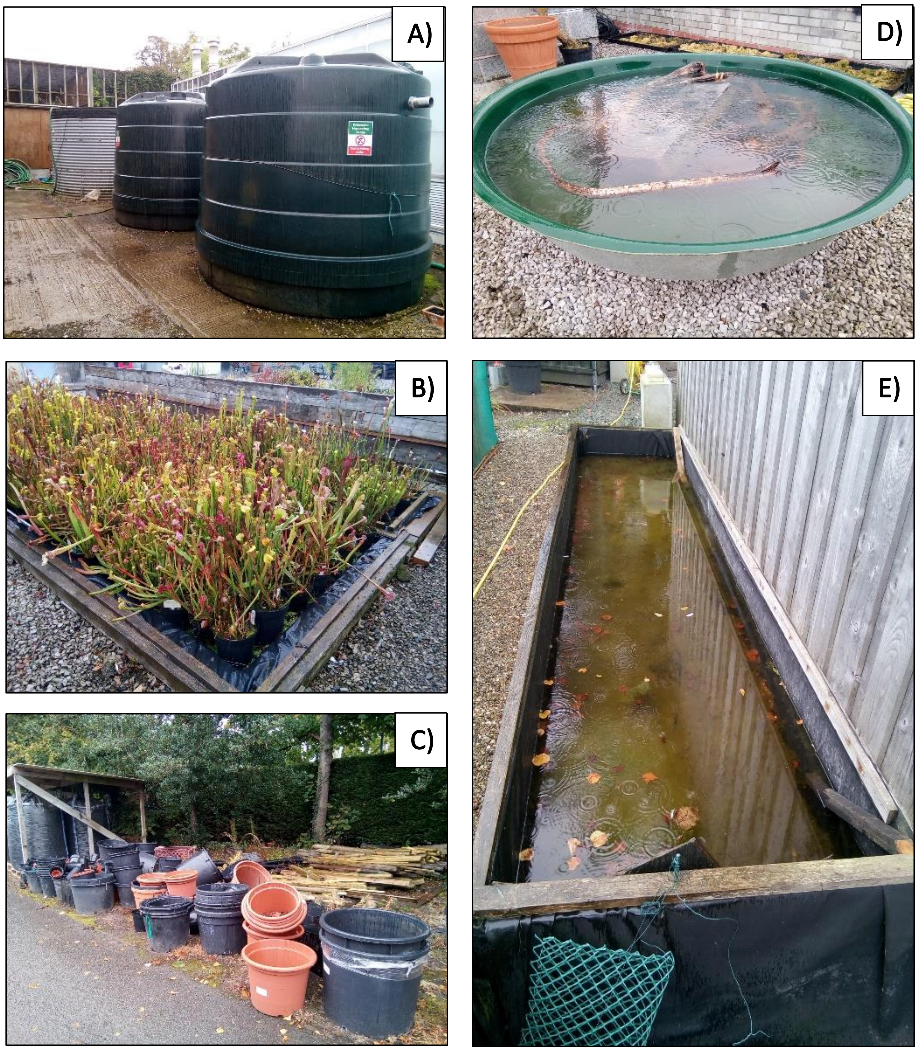

Figure S2. Water containers examined for immature mosquitoes. A) Rainwater containers; B) Water beds for damp soil plants; C) Unused plant pots; D) and E) Unused water beds for damp soil plants. Immature mosquitoes were only found in C).
